# Increased Regenerative Capacity of the Olfactory Epithelium in Niemann–Pick Disease Type C1

**DOI:** 10.3390/ijms18040777

**Published:** 2017-04-06

**Authors:** Anja Meyer, Andreas Wree, René Günther, Carsten Holzmann, Oliver Schmitt, Arndt Rolfs, Martin Witt

**Affiliations:** 1 Institute of Anatomy, University of Rostock, 18057 Rostock, Germany; anja.meyer@med.uni-rostock.de (A.M.); andreas.wree@med.uni-rostock.de (A.W.); ren.guenther@gmx.de (R.G.); schmitt@med.uni-rostock.de (O.S.); 2 Institute of Medical Genetics, Rostock University Medical Center, 18057 Rostock, Germany; carsten.holzmann@med.uni-rostock.de; 3 Albrecht-Kossel Institute for Neuroregeneration, Rostock University Medical Center, 18147 Rostock, Germany; arndt.rolfs@med.uni-rostock.de

**Keywords:** immunohistochemistry, cyclodextrin, allopregnanolone, miglustat, NPC1, mouse model, olfactory mucosa, bromodeoxyuridine, olfactory marker protein

## Abstract

Niemann–Pick disease type C1 (NPC1) is a fatal neurovisceral lysosomal lipid storage disorder. The mutation of the NPC1 protein affects the homeostasis and transport of cholesterol and glycosphingolipids from late endosomes/lysosomes to the endoplasmic reticulum resulting in progressive neurodegeneration. Since olfactory impairment is one of the earliest symptoms in many neurodegenerative disorders, we focused on alterations of the olfactory epithelium in an NPC1 mouse model. Previous findings revealed severe morphological and immunohistochemical alterations in the olfactory system of *NPC1*^−/−^ mutant mice compared with healthy controls (*NPC1*^+/+^). Based on immunohistochemical evaluation of the olfactory epithelium, we analyzed the impact of neurodegeneration in the olfactory epithelium of *NPC1*^−/−^ mice and observed considerable loss of mature olfactory receptor neurons as well as an increased number of proliferating and apoptotic cells. Additionally, after administration of two different therapy approaches using either a combination of miglustat, 2-hydroxypropyl-β-cyclodextrin (HPβCD) and allopregnanolone or a monotherapy with HPβCD, we recorded a remarkable reduction of morphological damages in *NPC1*^−/−^ mice and an up to four-fold increase of proliferating cells within the olfactory epithelium. Numbers of mature olfactory receptor neurons doubled after both therapy approaches. Interestingly, we also observed therapy-induced alterations in treated *NPC1*^+/+^ controls. Thus, olfactory testing may provide useful information to monitor pharmacologic treatment approaches in human NPC1.

## 1. Introduction

Neurodegenerative diseases become increasingly important because of an aging society. Olfactory impairment is one of the first symptoms in many neurodegenerative diseases and partly precedes motor symptoms for years. For example, 96% of patients with Parkinson’s disease demonstrate olfactory malfunction up to four years before the clinical onset of motor symptoms [1,2,3]. Alzheimer’s disease is also associated with a progressive olfactory impairment in about 90% of the patients [4,5]. Similar deficits are known for Huntington’s disease and other hereditary ataxias [6].

Although olfactory impairment has a direct influence on the patient’s quality of life and is frequently associated with neurodegenerative diseases, the olfactory system and in particular the olfactory epithelium has not been in the focus of extensive research activities [7,8]. Even in aged humans, the olfactory epithelium represents a neurogenic niche that replaces olfactory receptor neurons continuously throughout life, which allows the epithelium to recover after damage [9,10,11]. Regulation of olfactory neurogenesis is a multifaceted chain of molecular events leading to adequate proliferation and further differentiation of basal progenitor cells upon challenges of homeostasis during developing neurodegenerative diseases [12,13].

Previous studies of the olfactory system in a mouse model of Niemann–Pick disease type C1 (NPC1) reported a significant loss of olfactory receptor neurons in the olfactory epithelium as well as massive astrogliosis and microgliosis of the olfactory bulb [14,15]. NPC1 is an autosomal recessive lysosomal lipid storage disease with a progressive neurodegenerative and so far fatal course, which is caused by a mutation of the *NPC1* gene in 95% or of *NPC2* gene in 5% of the patients. This defect leads to a disturbed transport of different lipids from late endosomes/lysosomes to the endoplasmic reticulum and consequently to a toxic accumulation of unesterified cholesterol, glycolipids, glycosphingolipids (GSLs) and fatty acids [16,17,18,19,20]. The neuropathology is characterized by a progressive loss of Purkinje cells in the cerebellum [21,22,23,24,25] and neurons in other parts of the brain, e.g., basal ganglia, thalamus [26,27,28], piriform cortex, and hippocampus [27]. Further on, NPC1 is associated with disturbance of myelin integrity [26,29,30,31] and an almost complete absence of myelin in the corpus callosum [32].

Thus far, a substrate reduction therapy (SRT) with miglustat is the only already clinically permitted treatment in Europe. This small imino sugar reversibly inhibits the glycosylceramide synthase and reduces the accumulation of toxic metabolites like sphingomyelin, sphingosine and other complex GSLs and delayed the onset of neurological symptoms [20,33,34]. Former studies also proved the delay of neurological symptoms and a reduced hepatic cholesterol concentration after treatment with cyclodextrin (2-hydroxypropyl-β-cyclodextrin, HPβCD) [34,35,36]. HPβCD is a cyclic oligosaccharide that is thought to transfer cholesterol out of the lysosomes [37,38] and is, based on this promising effect, part of a phase 2b/3 prospective, randomized trial sponsored by Vtesse Inc. (Gaithersburg, MD, USA) [39]. A combination of SRT with a byproduct replacement therapy (BRT) with HPβCD and allopregnanolone demonstrated a delay in the onset of clinical symptoms, a pronounced increase of lifespan and a significant reduction of intracellular lipid accumulation as well as a prevention of the cerebellar Purkinje cell loss [21,38,40].

Since chemosensory systems were almost spared from NPC1 related studies, we used a 5-bromo-2′-deoxyuridine (BrdU) protocol and appropriate immunohistochemical markers, to quantify the impact of NPC1 on the regenerative capacity, apoptotic activity and number of mature olfactory receptor neurons of the olfactory mucosa in an NPC1 mouse model. Additionally, we analyzed the potential of two different therapeutic approaches using the combination of SRT and BRT with miglustat, HPβCD and allopregnanolone (COMBI) and a monotherapy with HPβCD.

## 2. Results

### 2.1. Histology of the Olfactory Mucosa

**Figure 1:**
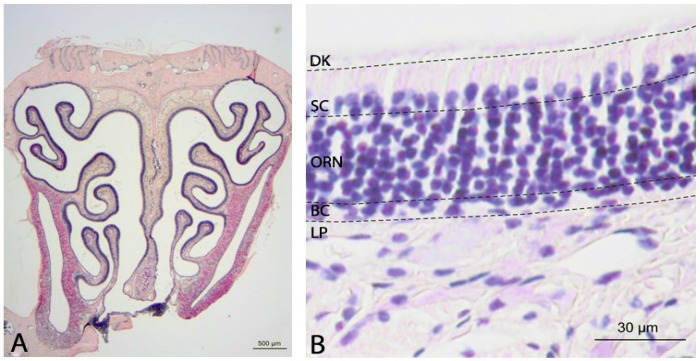
Structural overview of the OE (H&E): (**A**) frontal section of the nasal cavity of a *NPC1*^+/+^ mouse (56 days); and (**B**) structural composition of the olfactory mucosa including the lamina propria (LP) and the olfactory epithelium with basal cells (BC), olfactory receptor neurons (ORN), supporting cells (SC) and cilia/dendritic knobs (DK).

Sections were stained with hematoxylin and eosin (H&E) with an interval of 500 µm. A normal olfactory epithelium (OE) is composed of three different layers (Figure 1). The basal layer consists of horizontal and globose basal cells that act as stem and progenitor cells and are responsible for the regeneration of olfactory receptor neurons (ORNs). The medial part comprises several series of ORNs that end up in an apical dendritic knob with several cilia containing olfactory receptors, and basally in their axons connecting to the olfactory bulb. The third cell type includes the self-renewing supporting cells in the luminar layer of the OE.

**Figure 2:**
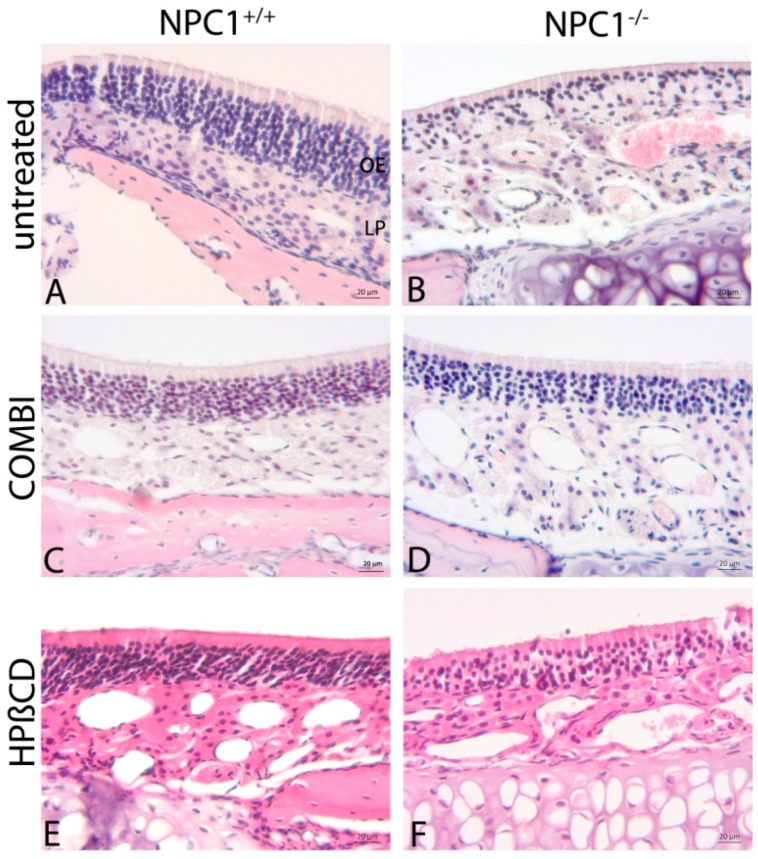
H&E staining of the OE: (**A**) Untreated *NPC1*^+/+^ demonstrate a typical columnar structure and a large number of ORNs; (**B**) Untreated *NPC1*^−/−^ exhibit massive morphological changes by a loss of regular layering and a markedly decreased cell density. COMBI-treated *NPC1*^−/−^ (**D**); and HPβCD-treated *NPC1*^−/−^ (**F**) demonstrate a significant improvement of the morphology and are barely distinguishable from their controls. COMBI-*NPC1*^+/+^ (**C**); and HPβCD-*NPC1*^+/+^ (**E**) exhibit a normal profile.

Light microscopy showed distinct morphological alterations of the *NPC1*^−/−^ OE (Figure 2). Whereas *NPC1*^+/+^ demonstrated the typical columnar arrangement of ORNs, it was hardly maintained in *NPC1*^−/−^. Instead, there were obvious interruptions in the continuity of ORNs and basal cells (Figure 2B) and an apparently decreased cell density.

Both treatment approaches showed a clear benefit for preservation of the regular morphology (Figure 2D,F). It was hardly possible to distinguish between treated *NPC1*^−/−^ and treated or untreated *NPC1*^+/+^ (Figure 2C,E) when observers were blinded.

### 2.2. Quantification of BrdU(+) Proliferating Cells

**Figure 3:**
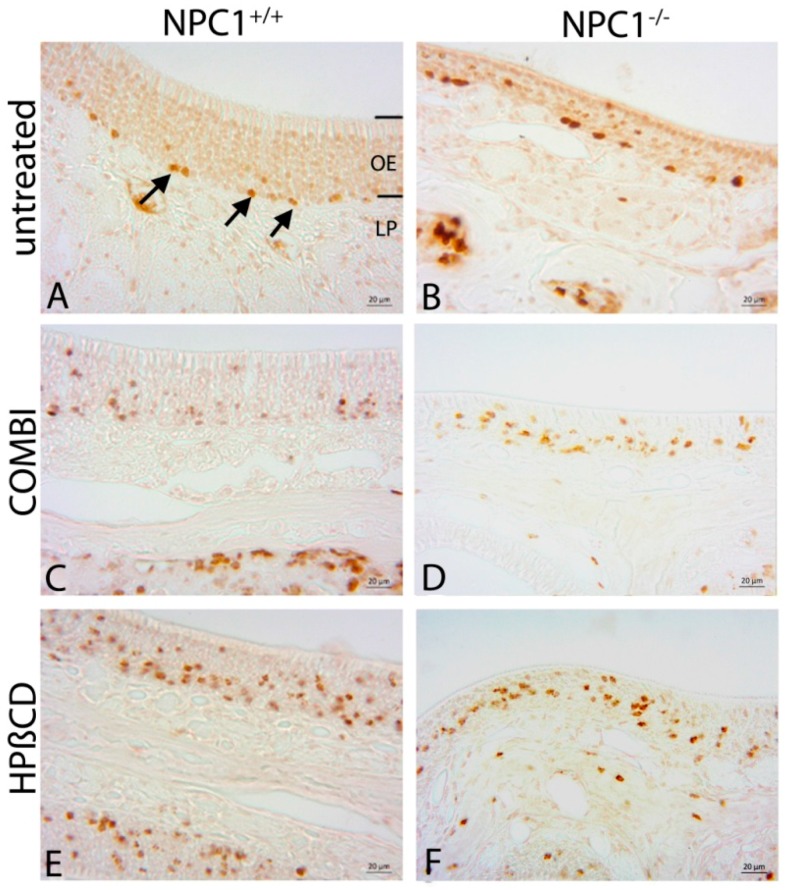
Immunohistochemical reaction of BrdU(+) proliferating cells. (**A**) Proliferating cells of untreated *NPC1*^+/+^ mice are mainly located in the basal third of the OE (arrows); (**B**) In untreated *NPC1*^−/−^, BrdU(+) cells are distributed in clusters in the basal compartment as well as in the region of ORNs and supporting cells. The thickness of the epithelium is considerably reduced. COMBI-treated *NPC1*^−/−^ (**D**); and HPΒCD-treated *NPC1*^−/−^ (**F**) demonstrate an increased proliferation activity with less clustered but more regularly distributed cells. COMBI-*NPC1*^+/+^ (**C**); and HPΒCD-*NPC1*^+/+^ (**E**) exhibit a higher proliferation activity than untreated *NPC1*^+/+^ (**A**) as well as untreated and treated *NPC1*^−/−^ (**B**,**D**,**F**) mice in all epithelial layers.

BrdU(+) proliferating cells were distributed in the entire OE in the layer of basal cells, ORNs and supporting cells (Figure 3). In *NPC1*^+/+^ animals, proliferating cells were mainly located and arranged like a string of pearls in the basal third of the OE. Occasionally, proliferating cells also occurred in the area of ORNs and supporting cells. In contrast, in *NPC1*^−/−^ OE, BrdU(+) cells were distributed in clusters in the basal compartment and additionally abundant in the region of supporting cells and ORNs.

Both treatment schedules seemed to upregulate the proliferation activity in *NPC1*^−/−^. Contrary to the untreated *NPC1*^−/−^ mice, the cells were distributed more regularly and not that clustered. However, they were further located in all, basal, medial and luminal layers of the OE. There was a similar tendency in both treated *NPC1*^+/+^ mouse groups. Treatments seemed to increase the proliferation and regeneration activity of basal cells, ORNs and supporting cells indicating that both treatments influenced and interfered the homeostasis of the OE in healthy *NPC1*^+/+^.

**Figure 4:**
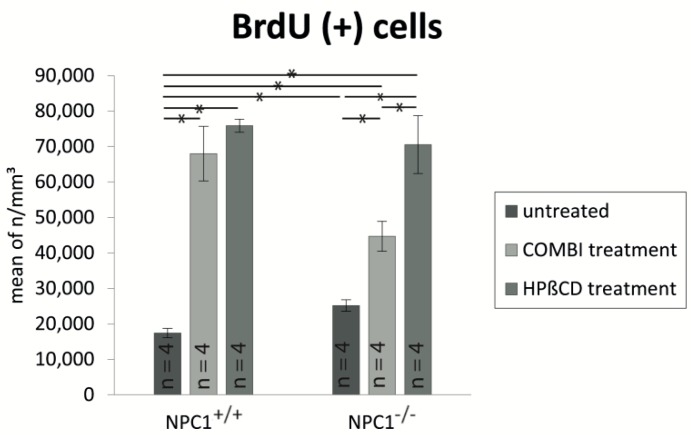
Proliferation analysis of untreated, COMBI- and HPΒCD-treated *NPC1*^+/+^ and *NPC1*^−/−^ mice show a remarkable increase of newly formed cells in all groups compared to untreated *NPC1*^+/+^. Both treatments result in a particularly clear enhancement of proliferation in *NPC1*^−/−^ but also in *NPC1*^+/+^. The monotherapy reveals a higher proliferation rate than the COMBI-treatment in *NPC1*^+/+^ and *NPC1*^−/−^ mice. All data represent the mean ± SEM. *p* < 0.05 was considered significant (* *p* < 0.05). For *p*-values see text.

For a more detailed distinction of the proliferation activity we quantified BrdU(+) cells of all six groups. We defined the number of BrdU(+) cells per mm^3^ of untreated *NPC1*^+/+^ 100% with 17,417 ± 1317 cells/mm^3^ and compared the cell densities. The proliferation in all other groups was significantly higher than in the control group (Figure 4). *NPC1*^−/−^ mice showed a significant increase of BrdU(+) cells with 145% (25,180 ± 1605 cells/mm^3^, *p* = 0.021), whereas both treatment approaches resulted in severe proliferation enhancement with 157% more BrdU(+) cells in COMBI-treated *NPC1^−/−^* mice (44,693 ± 4191 cells/mm^3^, *p* = 0.021) and 305% in HPΒCD-treated *NPC1^−/−^* mice (70,558 ± 8159 cells/mm^3^, *p* = 0.021). A comparison of *NPC1*^−/−^ mice showed a significant proliferation increase in these animals after both therapy approaches. Whereas COMBI-treated *NPC1*^−/−^ mice showed a significant increase of 19,513 cells/mm^3^ (~77.5%, *p* = 0.021) of newly formed cells, the monotherapy resulted in a further enhancement of 45,378 cells/mm^3^ (~180%) when compared with untreated *NPC1*^−/−^ mice (*p* = 0.021). Surprisingly, the proliferation increase was not only restricted to *NPC1*^−/−^ mice, but occurred also in treated *NCP1*^+/+^ groups. We detected an almost fourfold therapy-induced proliferation increase with 390% in COMBI-treated *NPC1*^+/+^ mice (67,972 ± 7694 cells/mm^3^, *p* = 0.021) and 436% in HPΒCD-treated *NPC1^+/+^* mice (75,871 ± 1865 cells/mm^3^, *p* = 0.021). The monotherapy with HPβCD induced approximately 58% more proliferating cells than combination therapy in *NPC1*^−/−^ mice (*p* = 0.021). Treated *NPC1^+/+^* mice showed a similar tendency, but without any statistical significance.

### 2.3. Quantification of OMP(+) Mature ORNs

**Figure 5:**
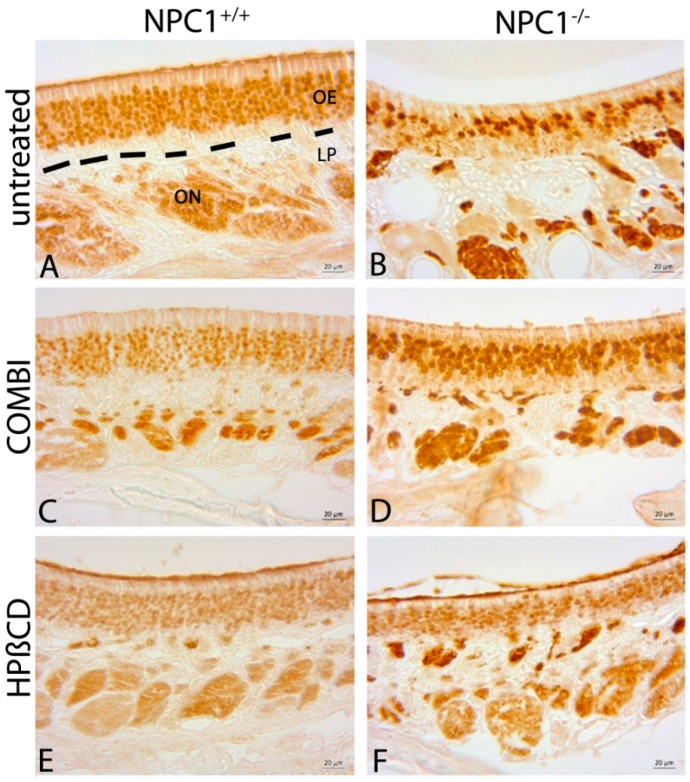
Immunohistochemical reaction of OMP(+) mature ORNs. (**A**) Untreated *NPC1*^+/+^ show a thick layer of highly organized mature ORNs; (**B**) Untreated *NPC1*^−/−^ exhibit only a few remaining disarranged mature ORNs in the upper third of the OE. COMBI-treated *NPC1*^−/−^ (**D**); and HPΒCD-treated *NPC1*^−/−^ (**F**) demonstrate a pronounced layer of preserved or already replaced mature ORNs. Nevertheless, the basal layer cannot be clearly differentiated and cells seem to be less organized. The reactivity of: OMP(+) ORNS in COMBI-*NPC1*^+/+^ (**C**); and HPΒCD-*NPC1*^+/+^ (**E**) do not show any alterations in comparison to untreated *NPC1*^+/+^ (**A**). In (**A**), the OE is demarcated by dotted lines from the lamina propria (LP). ON, olfactory nerve bundles in the LP.

Olfactory marker protein (OMP) is a marker for mature olfactory receptor neurons (ORNs) and possibly involved in the olfactory signal transduction pathway [41,42]. The olfactory epithelium of *NPC1*^+/+^ mice showed a dense layer of highly organized OMP(+) mature ORNs, which were clearly distinguishable from supporting and basal cells (Figure 5A). In contrast to the anti-BrdU labeling, treatments of *NPC1*^+/+^ mice did not result in immunohistochemical differences of OMP(+) pattern (Figure 5C,E). In contrast, *NPC1*^−/−^ mice displayed a considerable reduction of mature ORNs (Figure 5B). The few remaining mature ORNs were mainly located in the upper third of the OE, which appeared clearly thinner. The typical columnar arrangement was replaced by irregular cell arrangement and large gap-like spaces. Both treatments showed a notable benefit for the morphology in *NPC1*^−/−^. The OE revealed a pronounced layer of either preserved and/or already replaced mature ORNs. In contrast to *NPC1*^+/+^, the basal layer cannot be clearly distinguished and the ORNs seemed to be less organized (Figure 5D,F). Light microscopy did not allow distinguishing between the beneficial effect on the recovery and preservation of either therapy approach.

**Figure 6:**
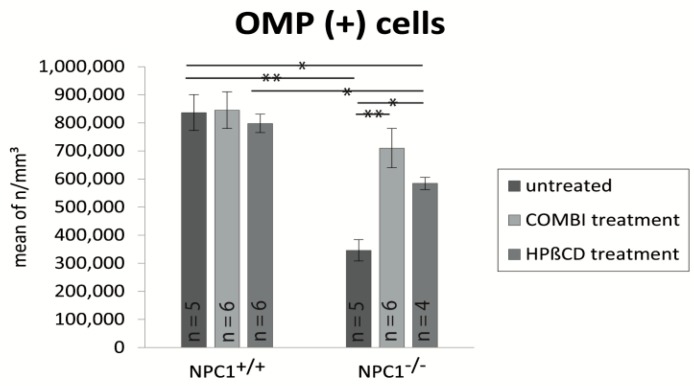
Analysis of mature OMP(+) ORNs of untreated, COMBI- and HPΒCD-treated *NPC1*^+/+^ and *NPC1*^−/−^ mice. Compared to the untreated controls, untreated *NPC1*^−/−^ mice show a significant reduction of mature ORNs that can be partly compensated by both therapies. However, COMBI-*NPC1*^−/−^ benefit more than their HPΒCD-treated conspecifics. In contrast, both treatments have no effect on the number of mature ORNs in *NPC1*^+/+^. All data represent the mean ± SEM. *p* < 0.05 was considered significant (* *p* < 0.05, ** *p* < 0.01). For *p-*values, see text.

Furthermore, we quantified the OMP(+) mature ORNs and estimated the cell density within the OE (Figure 6). Based on the OMP(+) cell density of 100% (836,392 ± 63,784 cells/mm^3^) in untreated *NPC1*^+/+^ mice, the quantity of mature ORNs in untreated *NPC1*^−/−^ mice was significantly reduced to 41% (346,129 ± 37,812 cells/mm^3^, *p* = 0.009). Both treatment schedules resulted in a distinct increase of OMP(+) cells with 85% in COMBI-treated *NPC1*^−/−^ mice (709,729 ± 69,558 cells/mm^3^, *p* = 0.100) and 70% in HPΒCD-treated *NPC1*^−/−^ mice (594,852 ± 43,952 cells/mm^3^, *p* = 0.014), but did not completely compensate for the neuronal loss at the *NPC1*^+/+^ level. COMBI-treated *NPC1*^−/−^ showed 363,600 cells/mm^3^ (~105%, *p* = 0.006) and HPΒCD-treated *NPC1*^−/−^ 238,139 cells/mm^3^ (~69%, *p* = 0.014) more OMP(+) cells than untreated *NPC1*^−/−^, indicating that the combination therapy had a bigger impact on the preservation of mature ORNs. However, the differences between both treatment approaches were not statistically significant.

### 2.4. Quantification of Cas-3(+) Apoptotic Cells

**Figure 7:**
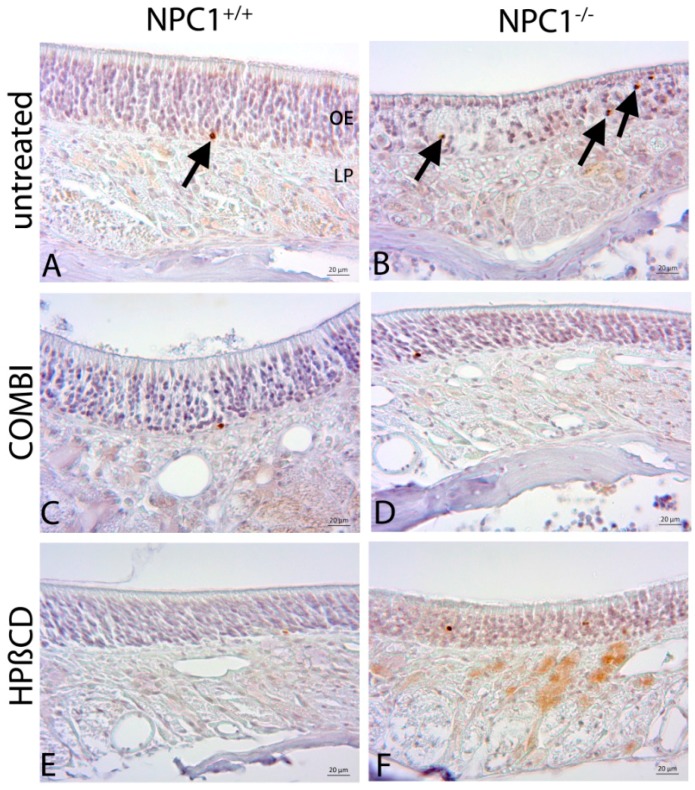
Immunohistochemical reaction of cas-3(+) apoptotic cells. Untreated *NPC1*^+/+^ (**A**) show occasionally apoptotic cells (arrow), whereas untreated *NPC1*^−/−^ (**B**) demonstrate a clear increase of frequently clustered cas-3(+) cells in the area of basal cells and ORNs. In comparison to the untreated *NPC1*^−/−^ (**B**); COMBI-treated *NPC1*^−/−^ (**D**); and HPΒCD-treated *NPC1*^−/−^ (**F**) exhibit a reduction of apoptosis. Light microscopic evaluation do not show any noticeable alterations in: COMBI-*NPC1*^+/+^ (**C**); and HPΒCD-*NPC1*^+/+^ (**E**); in comparison to the untreated *NPC1*^+/+^ (**A**).

Cas-3(+) apoptotic cells of untreated *NPC1*^+/+^ mice were found in ORN and basal cell layers but not in the layer of supporting cells (Figure 7A). On the contrary, *NPC1*^−/−^ animals demonstrated a clear increase of apoptotic cells, which occurred in regular intervals, frequently in clusters in the layer of ORNs as well as basal cells (Figure 7B). Cas-3(+) cells were not detected in the layer of supporting cells. The monotherapy with HPβCD in *NPC1*^−/−^ mice seems to lead to a higher apoptotic level than the combination therapy. A similar tendency could be observed in treated *NPC1*^+/+^ mice. COMBI-treated *NPC1*^+/+^ mice did not differ from untreated *NPC1*^+/+^ animals. However, HPΒCD-treated *NPC1*^+/+^ presented widely spaced but regularly cas-3(+) cells. 

**Figure 8:**
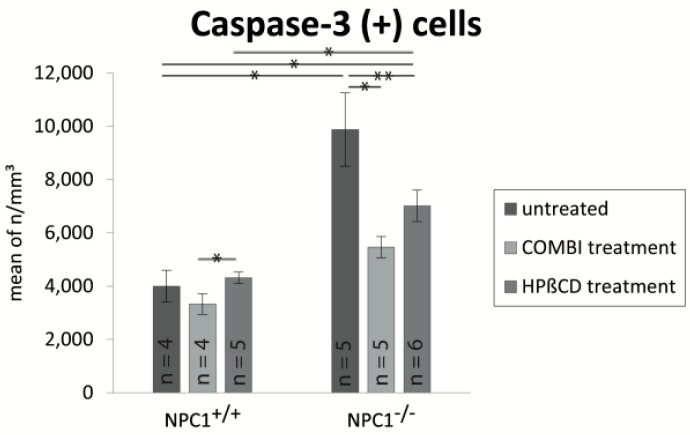
Analysis of cas-3(+) apoptotic cells of untreated, COMBI- and HPΒCD-treated *NPC1*^+/+^ and *NPC1*^−/−^ mice. Compared to the untreated controls, untreated *NPC1*^−/−^ mice show a significant increase of apoptosis. Both therapies compensate this effect up to a certain extend but cannot normalize it to the *NPC1*^+/+^ level. The cas-3 rates of COMBI- and HPΒCD-treated *NPC1*^+/+^ do not significantly differ from untreated *NPC1*^+/+^. However, HPΒCD-treated *NPC1*^+/+^ and *NPC1*^−/−^ reveal a higher apoptosis activity than their COMBI-treated conspecifics. All data represent the mean ± SEM. *p* < 0.05 was considered significant (* *p* < 0.05, ** *p* < 0.01). For *p*-values see text.

For a clear identification of relative cell density differences, we quantified cas-3(+) in all groups (Figure 8). Based on a cell density of approximately 4000 apoptotic cells/mm^3^ in *NPC1*^+/+^ mice, we detected an almost 2.5-fold significant increase of cas-3(+) cells in untreated *NPC1*^−/−^ mice (9879 ± 1373 cells/mm^3^, *p* = 0.014). In addition, after both treatment approaches, *NPC1*^−/−^ animals still showed higher apoptotic cell numbers than their controls with an increase of 37% in COMBI-treated *NPC1*^−/−^ (5471 ± 397 cells/mm^3^, *p* = 0.142) and 75% in HPΒCD-treated *NPC1*^−/−^ (7016 ± 591 cells/mm^3^, *p* = 0.019).

However, both therapies reduced apoptotic cell death in *NPC1*^−/−^ significantly when compared with untreated *NPC1*^−/−^, as the number of apoptotic cells decreased by 4408 cells/mm^3^ (~55%) in COMBI-treated *NPC1*^−/−^ (*p* = 0.009) and by 2863 cells/mm^3^ (~71%) in HPΒCD-treated *NPC1*^−/−^ (*p* = 0.045). In contrast, both treatments had no significant effect on apoptotic activity in *NPC1*^+/+^ mice with 83% (3327 ± 387 cells/mm^3^, *p* = 0.773) in COMBI-treated *NPC1*^+/+^ and 108% (4316 ± 215 cells/mm^3^, *p* = 0.327) in HPΒCD-treated *NPC1*^+/+^. Although we did not observe a significant difference between both treatment approaches in *NPC1*^−/−^, there was a significant increase of 989 cells/mm^3^ (~29%) of apoptotic cells in HPΒCD-treated *NPC1*^+/+^ when compared with COMBI-treated *NPC1*^+/+^ mice (*p* = 0.050) (Table 1).

### 2.5. Cathepsin D

**Figure 9:**
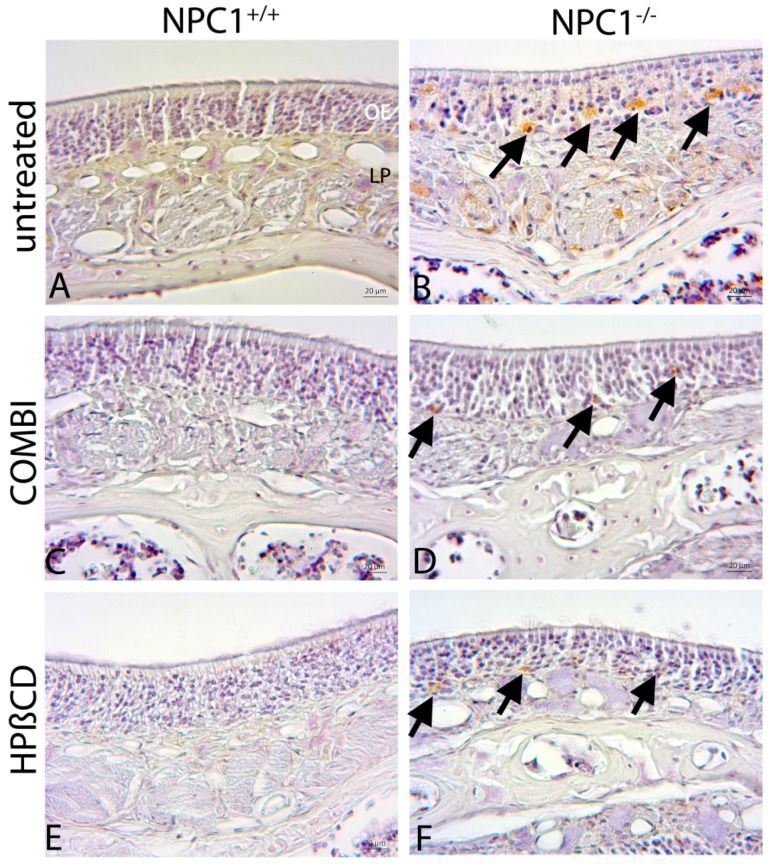
Immunohistochemical reaction of the olfactory mucosa revealed that cathD was negligible in untreated *NPC1*^+/+^ (**A**); COMBI-*NPC1*^+/+^ (**C**); and HPΒCD-*NPC1*^+/+^ (**E**). In contrast, untreated *NPC1*^−/−^ (**B**) exhibited a clear immunoreactivity of cathD within large inclusions in regularly distributed cells, mainly located in the basal layer (arrows). In COMBI-treated *NPC1*^−/−^ (**D**); and HPΒCD-treated *NPC1*^−/−^ (**F**), cathD(+) cells still occurred in regular intervals, but less frequently and in reduced size (arrows). There were no obvious differences between both therapies.

Cathepsin D (cathD), a marker for lysosomal activity, was strongly expressed in macrophage-like cells within the OE of untreated *NPC1^−/−^* animals (Figure 9). In contrast, cathD immunoreactivity was almost absent in both, untreated and treated *NPC1^+/+^* animals. Both treatments resulted in a reduction of cathD immunoreactivity in *NPC1^−/−^.* CathD(+) cells still occurred in regular intervals, but less frequently and in reduced size. A difference between the cathD immunoreactivities was not apparent in the treatment groups.

## 3. Discussion

Even though olfactory impairment is a known symptom and appears early in the course of neurodegenerative diseases like Alzheimer’s disease [43,44,45] or Parkinson’s disease [46,47], less is reported about the impact of neurodegeneration and regeneration on the olfactory epithelium. Adult neurogenesis occurs only in distinct regions of the mammalian organism, including the peripheral and central olfactory system, and consequently a steady turnover of neuroepithelial cells in the OE is maintained [48]. In this study, we analyzed the regenerative capacity of the OE in an *NPC1*^−/−^ mouse model and the impact of two different therapy approaches with a combination of miglustat, 2-hydroxypropyl-β-cyclodextrin (HPβCD) and allopregnanolone (COMBI) as well as a monotherapy with HPβCD. Therefore, we determined the number of BrdU(+) proliferating cells, OMP(+) mature ORNs, cas-3(+) apoptotic cells and, and assessed additionally, the occurrence of lysosomal activity with cathD in the different groups.

For the first time, we revealed the extent of neurodegeneration in the OE of *NPC1*^−/−^ in detail and demonstrated that the number of mature ORNs in eight-week-old *NPC1*^−/−^ mice was more than halved, whereas the caspase-dependent apoptotic activity has increased by more than 2.5 times. Despite an increased proliferation, the neuronal loss could not be fully compensated. Light microscopic evaluation of BrdU(+) cells revealed that the newly formed cells are distributed in the entire OE in the layers of basal cells, ORNs and supporting cells, indicating that the mitotically active basal cells already differentiated into ORNs and that proliferation also affected the self-renewing supporting cells. Furthermore, we detected a distinct enhancement of the lysosomal activity with cathD.

Additionally, we presented the beneficial effect of two symptomatic therapies on the pathology in the OE by a significantly increased proliferation activity and a clearly visible delay of morphologic degeneration. However, therapeutic effects are limited and the pathologic picture could not be normalized.

Surprisingly, both therapies resulted in a significant increase of the proliferation in *NPC1*^+/+^ animals. This effect was not reflected by quantitative alterations of the mature ORNs and could not be adequately explained by accompanying apoptotic processes. Although both treatment schedules worked with HPβCD, we detected significant differences between both therapies.

### 3.1. Olfactory Epithelium in NPC1^+/+^ Control Animals

Our results demonstrate the homeostasis of proliferation and apoptosis in the normal OE, in which mature ORNs represent a majority of the cells. CathD was negligible in both treated and untreated *NPC1*^+/+^.

Thus far, no data were available on the total number of ORNs in mice. For the first time, Kawagishi et al. (2014) published a quantification method for ORNs in a C57BL/6J mice model and estimated a total number of OMP(+) receptor neurons in the unilateral main olfactory epithelium with 5,140,000 ± 380,000 in males and 5,210,000 ± 380,000 in females with no significant differences between the sexes [49]. We adapted this method for the quantification of OMP(+) receptor neurons and cas-3(+) apoptotic cells in the unilateral OE of NPC1 and analyzed the impact of the disease and both treatment approaches on these cells. We estimated the mean value of the total number of OMP(+) ORNs in the unilateral OE without the OB of healthy *NPC1*^+/+^ mice 4,676,983 ± 184,344. These data indicate that both quantifications are comparable.

### 3.2. Morphological Alterations in NPC1^−/−^ Animals

We showed that, in the OE of week-week-old *NPC1*^−/−^ mice, the number of BrdU(+) cells was significantly increased, probably as a result of compensation of the severe loss of mature ORNs. While *NPC1*^−/−^ mice need to replace numerous dying neurons, there is less turnover in *NPC1*^+/+^ animals that has to be compensated. Our results reflect the constant ability of recovering processes after neuronal loss to maintain the integrity of olfactory processes [13].

Our previous findings showed a considerable reduction of axonal inputs of the ORNs (OMP(+) cells towards the olfactory bulb in eight-week-old *NPC1*^−/−^ mice [14]. The present data demonstrate in detail the extensive loss of mature ORNs. We detected a significant reduction of mature ORNs up to 41% indicating that the majority of the cell deficit affects these cells. Seo et al. (2014) confirmed these results and additionally found that the immature neuronal pool (GAP43(+) cells) was reduced in *NPC1*^−/−^ OE [15]. Supplementary, we detected a 2.5-fold significant increase of apoptotic cells proving the enormous neuronal loss in *NPC1*^−/−^. The increased immunohistochemical reactivity for cathD in *NPC1*^−/−^ mice confirms own previous studies on the involvement of lysosomal activity and the presence of galectin-3, a carbohydrate-binding protein that is released mainly by macrophages in the OE [14,50]. Increased levels of cathD are associated with neurodegeneration like in Alzheimer’s and Gaucher’s disease or LSDs and partially appear before the onset of major pathology [51,52,53,54]. Similar effects have also been described in NPC1 for brainstem, cerebellum, hippocampus and liver [18,50,55].

### 3.3. Combination Treatment of NPC1^−/−^ Mice Shows Beneficial Effects

NPC1 disease proceeds lethally and only symptomatic therapies are available. Earlier studies in the same murine model demonstrated a synergistic effect of the combination of miglustat, HPβCD and allopregnanolone, resulting in a significant reduction of GSL accumulation, a delay in onset of clinical signs, 30% increase of lifespan, rescue of cerebellar Purkinje cells, motor behavioral improvement and regression of corneal inclusions [21,34,56,57].

Our results confirm the beneficial effect on normalized motor behavior and prevention of weight loss. With regard to cell and tissue dynamics, our data revealed that the number of proliferating cells significantly increases. Furthermore, after seven weeks of treatment, the number of mature ORNs did not diminish in the same degree, and apoptotic processes were distinctly reduced to 55% when compared to untreated *NPC1*^−/−^. Accordingly, we observed a reduced immunoreactivity of cathD compared with untreated *NPC1*^−/−^, however, accompanied with an immunoreactivity still more extensive than in *NPC1*^+/+^ mice indicating ongoing neurodegenerative processes.

### 3.4. Monotherapy with HPβCD

Previously only used as a vehicle for allopregnanolone, monotherapy with HPβCD had distinct effects. For example, Tanaka et al. (2015) showed that weekly subcutaneous injections of up to 4000 mg/kg HPβCD improved the lifespan and significantly attenuated symptoms and liver cholesterol sequestration of *NPC1*^−/−^ mice. Additionally, they observed a benefit after treatment with 2500 mg/kg HPβCD twice a week in a patient with NPC [58]. Maarup et al. (2015) demonstrated a stabilization of a patient’s pathological state after 1.5 years of treatment with 200 mg intrathecally administered HPβCD [59]. Matsuo et al. (2013) also proved a benefit of HPβCD during the first six months after a gradually increasing treatment of 80 mg/kg to 2.5 g/kg in two patients. Even though they did not recognize any alterations of the neurological deficits, the treatment reduced the hepatosplenomegaly and central nervous dysfunction [60]. However, a major side effect of HPβCD is hearing loss [61,62,63]. Other possible cytotoxic effects of HPβCD at a relatively high dose cannot be ruled out, but Tanaka et al. (2015) [58] showed that comparisons between different HPβCD concentrations applied in *NPC1*^−/−^ mice did not indicate differences in putative toxicity (in a range between 1000 and 4000 mg/kg, applied subcutaneously); highest survival rates were achieved with a dose of 4000 mg/kg in mice, but the limitations of this and other cyclodextrin studies are the lack of other frequencies of administration [58]. In a recent study, Megias-Vericat et al. (2017) compiled adverse effects of HPβCD in 17 patients, 11 of which were related to the drug [64], however, attested a low toxic potential.

Our results show a beneficial effect with a significant increase of proliferation by 180% and mature ORNs rate by 69% as well as an up to 71% significantly reduced apoptotic activity when compared to the initial values of untreated *NPC1*^−/−^ mice. Similar results were found in the olfactory bulb of cyclosporin A treated *NPC1*^−/−^ mice [15]. Seo et al. (2014) detected 66% more DCX(+) cells, a neural precursor marker, in the glomerular layer and a 1.5-fold increase of BrdU labeled cells in the granule cell layer derived from the subventricular zone [15]. Similar to the COMBI-treated *NPC1*^−/−^ mice, treatment with HPβCD reduced the cathD reactivity but did not normalize it to the *NPC1*^+/+^ level. Cluzeau et al. [50] also investigated the impact of HPβCD and miglustat on cathD mRNA expression in *NPC1*^−/−^ liver and showed a normalization of cathD to the *NPC1*^+/+^ level after daily HPβCD treatment, whereas miglustat had only a moderate effect.

### 3.5. Limitations of Symptomatic Treatments

However, both treatment schedules showed limited benefit and did not completely prevent the neuronal loss in *NPC1*^−/−^. The usage of only one concentration of HPβCD may render it difficult to decide if certain effects on cellular homeostasis were caused by too high dosage or an optimal dose of HPβCD and/or miglustat.

Further on and despite an additional increase of proliferating cells of 78% in COMBI-treated *NPC1*^−/−^ and even 180% in HPΒCD-treated *NPC1*^−/−^ animals, compared to the untreated *NPC1*^−/−^, the number of mature ORNs is partly significantly reduced. Possibly, despite the increased proliferation there are still not enough newly formed cells that can compensate the loss of neurons. More probably, these cells failed to differentiate into mature ORNs, remained in any other stage of differentiation or even died [65]. Hinds et al. [66] examined the turnover of ORNs and suggested that most or all newly formed cells that fail to establish synapses with the olfactory bulb die. Our results of the apoptosis analyses confirm a still increased number of dying cells indicating that the apoptotic activity cannot be reduced to wildtype level despite the therapy. These newly formed cells can potentially influence the rate of non-neuronal cell lines like supporting cells or cells of different maturity level that are not detected with our immunohistochemical markers. Furthermore, the increased expression of cathD in *NPC1*^−/−^ could not be normalized after both treatment approaches. Our results demonstrated a reduction of cathD in *NPC1*^−/−^ OE after both treatments without any obvious differences. Possibly, the benefit is mainly due to HPβCD, but further investigations are needed.

### 3.6. Therapy-Induced Changes in NPC1^+/+^ Mice 

Surprisingly, the therapeutic effects are not restricted to *NPC1*^−/−^ mice but are also found in *NPC1*^+/+^ littermates. The proliferation in both treated *NPC1*^+/+^ groups is massively increased, but we could not record any corresponding increase of mature ORNs numbers. Both treatment approaches probably maximize the proliferation ability resulting in an up to more than fourfold increase of BrdU(+) cells. Possibly, wildtype mice have a less limited precursor cell reserve than *NPC1*^−/−^ mice, which may explain the differences between the genotypes. However, we could not detect alterations in apoptosis in *NPC1*^+/+^ after treatment. It might be that we did not detect the whole extent of apoptosis because of the very short caspase-dependent apoptotic processes during cell cycles. Moreover, we cannot exclude alterations in alternative mechanisms of cell death like necrosis or autophagy [67]. Finally, the fate of these newly formed cells is still unclear. However, miglustat and COMBI treatment of *NPC1*^+/+^ mice lead to decreased body weights, brain size and partly impaired performance in behavioral tests, possibly due to decrease of cholesterol and other lipids below normal level, interfering with normal cellular or membrane functions [68]. This would be in line with the observation that one of the major side effects of HPβCD administration resulted in ototoxicity, presumably by membrane dysfunction of the sensitive hair cells [62,69]. Considering olfactory homeostasis, further investigations on differential transcription factor expression that regulates OE neurogenesis [13] are necessary.

### 3.7. Differences between the Therapeutic Effects

The proliferation rate in HPΒCD-treated *NPC1*^−/−^ mice is significantly higher than in COMBI- treated *NPC1*^−/−^ mice, although both treatment approaches used HPβCD. It has been shown that ß-cyclodextrin triggers the activity of caspase-3 and caspase-7 of keratinocytes in vitro, and significantly increases LDH rates in concentrations of 0.5% (*w*/*v*) and higher, whereas concentrations up to 0.1% (*w*/*v*) do not show any antiproliferative influence on HaCaT keratinocytes rather than sometimes even proliferative effects [70]. These findings allow the assumption that the HPβCD monotherapy of NPC1^−/−^ mice led to a compensatory cell proliferation by triggering apoptosis of ORNs. Yokoo et al. [71] also investigated the impact of HPβCD on leukemic cell lines and even attempted to use the potentially apoptosis inducing effect. They demonstrated that HPβCD had anticancer effects by inhibiting leukemic cell growth and inducing apoptosis. Although our results show a 28% higher casp3 activity in HPΒCD-treated *NPC1*^−/−^ than the COMBI-treated *NPC1*^−/−^ mice, there is no statistical significance probably because of a too small number of samples. However, HPΒCD-treated *NPC1*^−/−^ have a proliferation rate similar to the treated *NPC1*^+/+^ controls, whereas COMBI-treated *NPC1*^−/−^ generate significantly less new cells. Pharmacologic interactions between these substances are not yet completely understood. It is possible that miglustat has an inhibitory or antagonistic effect in combination with HPβCD and allopregnanolone. In contrast to our results, Wang et al. [72] examined the therapeutic potential of allopregnanolone in rats and revealed a significantly increased neuroprogenitor cell and human neural stem cell proliferation. Zampieri et al. [73] described that allopregnanolone may protect cells from oxidative damage and from peroxide-induced apoptosis. Because oxidative stress triggers apoptosis, this effect could explain the lower proliferation rate in our COMBI- treated mice.

However, in terms of proliferation and mature ORN numbers we could not detect a difference between both therapies in *NPC1*^+/+^ mice. In contrast to the treated *NPC1*^−/−^, we observed a significant increase of almost 30% of apoptotic cells in HPΒCD-treated *NPC1*^+/+^ when compared with COMBI-treated *NPC1*^+/+^ animals, which supports the assumption that HPβCD may trigger apoptosis. It is likely that untreated *NPC1*^+/+^ mice already formed a maximum of mature ORNs. Consequently, even the increased proliferation cannot increase the number of OMP(+) cells in both treatment approaches. However, further investigations are necessary to clarify the fate of these numerous newly formed cells.

## 4. Methods

### 4.1. Animals

Heterozygous breeding pairs of NPC1 mice (BALB/cNctr-*Npc1*m1N/-J) were obtained from Jackson Laboratories (Bar Harbor, ME, USA) for generating homozygous *NPC1*^−/−^ mutants and *NPC1*^+/+^ control wild type mice. Mice were maintained under standard conditions with free access to food and water with a 12 h day/night cycle, a temperature of 22 °C and a relative humidity of 60%. Genotypes were determined until postnatal day P7 by PCR analysis. Eighteen NPC1 mutants and eighteen wild type controls of both sexes, aged up to eight weeks, were used for different therapeutic treatment schedules.

All animal procedures were approved by the Committee on the Ethics of Animal Experiments of the University of Rostock (approval ID: 7221.3-1.1-030/12, 14 June 2012).

### 4.2. Genotyping

For genotyping by PCR analysis, 1–2 mm of the tails were clipped at postnatal day P6 and homogenized in DirectPCR-Tail and 1% proteinase K (Peqlab, Erlangen, Germany) at 55 °C with 750 rpm for 16 h overnight on a Thermo Mixer (Eppendorf, Hamburg, Germany). Extracts were centrifuged for 30 s with 6000 rpm and PCR analysis was performed twice with 2 µL of the lysate and two different primer pairs under equal cycling conditions. For detecting the mutant allele (obtained fragment size 475 bp) primers 5′-GGTGCTGGACAGCCAAGTA-3′ and 5′-TGAGCCCAAGCATAACTT-3′ and for the wild type allele (obtained fragment size 173 bp) 5′-TCTCACAGCCACAAGCTTCC-3′ and 5′-CTGTAGCTCATCTGCCATCG-3′ were used.

### 4.3. Pharmacologic Treatment

**Figure 10:**
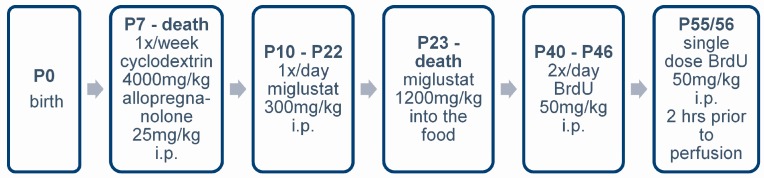
Scheme of the drug application for the combination treatment.

We used two different therapeutic schedules for the *NPC1*^−/−^ mutants and their *NPC1*^+/+^ controls. The first one was a combination treatment (COMBI) of synergistically working drugs utilizing HPβCD, allopregnanolone and miglustat, starting at postnatal day P7 with an injection of allopregnanolone (Pregnan-3α-ol-20-one; 25 mg/kg; Sigma Aldrich, St. Louis, MO, USA) dissolved in cyclodextrin (2-hydroxypropyl-β-cyclodextrin; 4000 mg/kg, i.p.; Sigma Aldrich, in Ringer’s solution) once a week, as described by [34]. Additionally, 300 mg/kg miglustat (*N*-butyl-deoxynojirimycin; generous gift of Actelion Pharmaceuticals, Allschwil, Schwitzerland) dissolved in normal saline solution was intraperitoneally injected daily from P10 to P22. Afterwards, miglustat powder was administered mixed with food (summarized in Figure 10). For the second treatment schedule, allopregnanolone and miglustat were omitted and only HPβCD was injected weekly. Controls included treated and untreated *NPC1*^+/+^ animals as well as untreated *NPC1*^−/−^ mutants.

### 4.4. BrdU Injections

BrdU (5-bromo-2′-deoxyuridine) is a thymidine analogue, which is incorporated in DNA during the S-phase of DNA synthesis. Consequently, it is a reliable marker for the quantification of the proliferative potential of tissues [74,75]. To label proliferating cells, all mice were intraperitoneally injected with BrdU (solubilized in normal saline, 50 mg/kg, Sigma, St. Louis, MO, USA) twice a day from P40 to P46. Additionally a final single dose was given 1 h before perfusion at P55–P56 for labeling the dividing cells of the olfactory epithelium.

### 4.5. Sample Preparation

Mice were deeply anesthetized with a mixture of 50 mg/kg ketamine hydrochloride (Bela-Pharm GmbH & Co KG, Vechta, Germany) and 2 mg/kg body weight of xylazine hydrochloride (Rompun; Bayer HealthCare, Leverkusen, Germany) and then intracardially perfused with normal saline solution, followed by 4% paraformaldehyde (PFA) in 0.1 M PBS. Mice were then decapitated, skinned, spare tissue was removed and the remaining skull including the nasal turbinates and the whole brain were post-fixed in 4% PFA for 24 h at 4 °C. Subsequently, heads were decalcified in 10% EDTA for 5–6 days at 37 °C, dehydrated and embedded in paraffin. The heads were serially cut in 10 µm in frontal direction from the tip of the nose to the anterior olfactory bulb and collected.

### 4.6. Immunohistochemistry

For the quantification of proliferating cells every 10th section (spaced 100 µm apart) was subjected to anti-BrdU immunohistochemistry (Abd Serotec, Puchheim, Germany). Sections were deparaffinized, rehydrated and pretreated with microwaves in 0.1 M citrate buffer (5 min, 680 W) followed by incubation with 3% H_2_O_2_ in PBS to block endogenous peroxidases for 30 min, and 5% normal goat serum (NGS) in PBS for 45 min to block nonspecific binding sites. Subsequently, sections were exposed to the primary antibody against BrdU (1:2000) in 3% NGS/PBS overnight at 4 °C. One section of each slide was used for negative control. After washing in PBS, the sections were sequentially incubated for 1 h with the secondary anti-rat IgG (1:200; Vector, Burlingame, CA, USA), the streptavidin-biotin-complex (ABC) reagent for 1 h (Vectastain-Elite; Vector, Burlingame, CA, USA) and finally visualized with H_2_O_2_—activated 3,-3,-diaminobenzidine (DAB, Sigma, Munich, Germany). Sections were dehydrated, mounted with DePeX and coverslipped.

For the quantification of mature ORNs adjacent sections were incubated similarly with antiserum against olfactory marker protein (OMP, Cat No. O7889, Sigma, St. Louis, MO, USA), and an additional series was incubated with anti-cleaved caspase-3 (cas-3, clone Asp175, Cat No. 9661, Cell Signaling Technology, Danvers, MA, USA) as well as anti-cathepsin D (Cat No. PU205-UP, BioGenex Laboratories, San Ramon, CA, USA). Pretreatment was carried out with microwaves in 0.1 M citrate buffer (5 min 850 W and 5 min 340 W). After blocking procedures, sections were exposed either to rabbit anti-OMP (1:6000 in blocking solution), or anti-caspase-3 (1:500) or anti-cathepsin D (1:6000) overnight at 4 °C and subsequently developed as described above. For controls, primary antisera were omitted. In negative controls no reactivity was observed.

### 4.7. Stereology and Statistic Evaluation

For the quantification of BrdU- positive cells of the OE, 32–40 complete sections of each mouse in units of averaged 5000 µm length were counted, using an unbiased stereological method, the optical fractionator. For each group and each genotype 4 animals were counted using a computer-aided microscope (Olympus BX-51, Hamburg, Germany) and a stereology software (Stereo Investigator v7.5, MBF Bioscience, Williston, ND, USA). The whole OE was first outlined using a 2× or 4× objective lens. Counting was realized at 40× magnification. The cell density of proliferating cells per mm^3^ of OE was averaged and the six different groups (summarized in Table 1) were compared. Therefore, the untreated mutants and untreated *NPC1*^+/+^ mice served as a reference for both COMBI-treated and HPΒCD-treated mice.

The cell densities of OMP(+) mature ORNs and apoptotic cas-3(+) cells were estimated as described before [49]. Briefly, the unilateral OE of the 4–5 sections with an interval of 1000 µm were traced using a lower magnification (4×) and labeled cells were counted with the 40× objective lens. For each group and genotype, 4–6 mice were analyzed.

Results are expressed as mean values ± SEM. Statistical evaluation was done with a Mann–Whitney *U*-test by SPSS (v.15.0.1, Chicago, IL, USA) using genotype and treatment group as independent variables. *p* < 0.05 was considered significant.

## 5. Conclusions

In summary, the olfactory neuroepithelium in *NPC1*^−/−^ mice reacts significantly with proliferation and almost complete reconstitution of its functional integrity after continuous COMBI treatment as well as after a monotherapy with HPβCD. First behavioral tests indicate concomitant functional olfactory acuity after respective therapies. Due to the constant and lifelong recovery of ORNs, olfactory testing may be a useful approach to monitor therapeutic management of NPC1 in humans. To underline the potential of olfactory acuity as a biomarker, we are currently performing behavioral sniffing tests in NPC1^−/−^ mice.

## Figures and Tables

**Table 1:** Summary of the mean values ± SEM in cells/mm^3^ of the cell densities of proliferating BrdU(+) cells, mature OMP(+) ORNs and apoptotic cas-3(+) for the six different treatment groups show distinct alterations depending on genotype and treatment option.

Treatment Group	BrdU (Cells/mm^3^ ± SEM)	OMP (Cells/mm^3^ ± SEM)	Cas-3 (Cells/mm^3^ ± SEM)
**untreated *NPC1^+/+^***	17,417 ± 1317	836,392 ± 63,784	3999 ± 596
**untreated *NPC1^−/−^***	25,180 ± 1605	346,129 ± 37,812	9879 ± 1373
**COMBI *NPC1^+/+^***	67,972 ± 7694	844,819 ± 65,313	3327 ± 387
**COMBI *NPC1^−/−^***	44,693 ± 4191	709,729 ± 69,558	5471 ± 397
**HPΒCD *NPC1^+/+^***	75,871 ± 1865	797,470 ± 32,568	4316 ± 215
**HPΒCD *NPC1^−/−^***	70,558 ± 8159	584,268 ± 21,134	7016 ± 591

## References

[1] Ross G.W., Petrovitch H., Abbott R.D., Tanner C.M., Popper J., Masaki K., Launer L., White L.R. (2008). Association of olfactory dysfunction with risk for future Parkinson’s disease. Ann. Neurol..

[2] Hawkes C.H., Shephard B.C., Daniel S.E. (1999). Is Parkinson’s disease a primary olfactory disorder?. QJM.

[3] Doty R.L., Deems D.A., Stellar S. (1988). Olfactory dysfunction in Parkinsonism: A general deficit unrelated to neurologic signs, disease stage, or disease duration. Neurology.

[4] Mesholam R.I., Moberg P.J., Mahr R.N., Doty R.L. (1998). Olfaction in neurodegenerative disease: A meta-analysis of olfactory functioning in Alzheimer’s and Parkinson’s diseases. Arch. Neurol..

[5] Djordjevic J., Jones-Gotman M., de Sousa K., Chertkow H. (2008). Olfaction in patients with mild cognitive impairment and Alzheimer’s disease. Neurobiol. Aging.

[6] Moberg P.J., Pearlson G.D., Speedie L.J., Lipsey J.R., Strauss M.E., Folstein S.E. (1987). Olfactory recognition: Differential impairments in early and late Huntington’s and Alzheimer’s diseases. J. Clin. Exp. Neuropsychol..

[7] Croy I., Nordin S., Hummel T. (2014). Olfactory disorders and quality of life—An updated review. Chem. Sens..

[8] Karpa M.J., Gopinath B., Rochtchina E., Jie Jin W., Cumming R.G., Sue C.M., Mitchell P. (2010). Prevalence and neurodegenerative or other associations with olfactory impairment in an older community. J. Aging Health.

[9] Loo A.T., Youngentob S.L., Kent P.F., Schwob J.E. (1996). The aging olfactory epithelium: Neurogenesis, response to damage, and odorant-induced activity. Int. J. Dev. Neurosci..

[10] Graziadei P.P., Monti Graziadei G.A. (1979). Neurogenesis and neuron regeneration in the olfactory system of mammals. I. Morphological aspects of differentiation and structural organization of the olfactory sensory neurons. J. Neurocytol..

[11] Carr V.M., Farbman A.I. (1993). The dynamics of cell death in the olfactory epithelium. Exp. Neurol..

[12] Mackay-Sim A., St John J., Schwob J.E., Doty R.L. (2015). Neurogenesis in the adult olfactory epithelium. Handbook of Olfaction and Gustation.

[13] Schwob J.E., Jang W., Holbrook E.H., Lin B., Herrick D.B., Peterson J.N., Hewitt Coleman J. (2017). Stem and progenitor cells of the mammalian olfactory epithelium: Taking poietic license. J. Comp. Neurol..

[14] Hovakimyan M., Meyer A., Lukas J., Luo J., Gudziol V., Hummel T., Rolfs A., Wree A., Witt M. (2013). Olfactory deficits in Niemann-Pick Pick type C1 (NPC1) disease. PLoS ONE.

[15] Seo Y., Kim H.-S., Shin Y., Kang I., Choi S.W., Yu K.-R., Seo K.-W., Kang K.-S. (2014). Excessive microglial activation aggravates olfactory dysfunction by impeding the survival of newborn neurons in the olfactory bulb of Niemann-Pick disease type C1 mice. Biochim. Biophys. Acta (BBA) Mol. Basis Dis..

[16] Chen F.W., Gordon R.E., Ioannou Y.A. (2005). NPC1 late endosomes contain elevated levels of non-esterified (’free’) fatty acids and an abnormally glycosylated form of the NPC2 protein. Biochem. J..

[17] Neufeld E.B., Wastney M., Patel S., Suresh S., Cooney A.M., Dwyer N.K., Roff C.F., Ohno K., Morris J.A., Carstea E.D. (1999). The Niemann-Pick C1 protein resides in a vesicular compartment linked to retrograde transport of multiple lysosomal cargo. J. Biol. Chem..

[18] Liao G., Yao Y., Liu J., Yu Z., Cheung S., Xie A., Liang X., Bi X. (2007). Cholesterol accumulation is associated with lysosomal dysfunction and autophagic stress in NPC1^−/−^ mouse brain. Am. J. Pathol..

[19] Sokol J., Blanchette-Mackie J., Kruth H.S., Dwyer N.K., Amende L.M., Butler J.D., Robinson E., Patel S., Brady R.O., Comly M.E. (1988). Type C Niemann-Pick disease. Lysosomal accumulation and defective intracellular mobilization of low density lipoprotein cholesterol. J. Biol. Chem..

[20] Te Vruchte D., Lloyd-Evans E., Veldman R.J., Neville D.C., Dwek R.A., Platt F.M., van Blitterswijk W.J., Sillence D.J. (2004). Accumulation of glycosphingolipids in Niemann-Pick C disease disrupts endosomal transport. J. Biol. Chem..

[21] Maass F., Petersen J., Hovakimyan M., Schmitt O., Witt M., Hawlitschka A., Lukas J., Rolfs A., Wree A. (2015). Reduced cerebellar neurodegeneration after combined therapy with cyclodextrin/allopregnanolone and miglustat in NPC1: A mouse model of Niemann-Pick type C1 disease. J. Neurosci. Res..

[22] Elleder M., Jirasek A., Smid F., Ledvinova J., Besley G.T. (1985). Niemann-Pick disease type C. Study on the nature of the cerebral storage process. Acta Neuropathol..

[23] Tanaka J., Nakamura H., Miyawaki S. (1988). Cerebellar involvement in murine sphingomyelinosis: A new model of Niemann-Pick disease. J. Neuropathol. Exp. Neurol..

[24] Higashi Y., Murayama S., Pentchev P.G., Suzuki K. (1993). Cerebellar degeneration in the Niemann-Pick type C mouse. Acta Neuropathol..

[25] Sarna J.R., Larouche M., Marzban H., Sillitoe R.V., Rancourt D.E., Hawkes R. (2003). Patterned Purkinje cell degeneration in mouse models of Niemann-Pick type C disease. J. Comp. Neurol..

[26] German D.C., Quintero E.M., Liang C.L., Ng B., Punia S., Xie C., Dietschy J.M. (2001). Selective neurodegeneration, without neurofibrillary tangles, in a mouse model of Niemann-Pick C disease. J. Comp. Neurol..

[27] Yamada A., Saji M., Ukita Y., Shinoda Y., Taniguchi M., Higaki K., Ninomiya H., Ohno K. (2001). Progressive neuronal loss in the ventral posterior lateral and medial nuclei of thalamus in Niemann-Pick disease type C mouse brain. Brain Dev..

[28] Love S., Bridges L.R., Case C.P. (1995). Neurofibrillary tangles in Niemann-Pick disease type C. Brain.

[29] Yan X., Lukas J., Witt M., Wree A., Hübner R., Frech M., Köhling R., Rolfs A., Luo J. (2011). Decreased expression of myelin gene regulatory factor in Niemann-Pick type C 1 mouse. Metab. Brain Dis..

[30] Goodrum J.F., Pentchev P.G. (1997). Cholesterol reutilization during myelination of regenerating pns axons is impaired in Niemann-Pick disease type C mice. J. Neurosci. Res..

[31] Yu T., Lieberman A.P. (2013). NPC1 acting in neurons and glia is essential for the formation and maintenance of cns myelin. PLoS Genet..

[32] German D.C., Liang C.L., Song T., Yazdani U., Xie C., Dietschy J.M. (2002). Neurodegeneration in the Niemann-Pick C mouse: Glial involvement. Neuroscience.

[33] Platt F.M., Jeyakumar M. (2008). Substrate reduction therapy. Acta Paediatr..

[34] Davidson C.D., Ali N.F., Micsenyi M.C., Stephney G., Renault S., Dobrenis K., Ory D.S., Vanier M.T., Walkley S.U. (2009). Chronic cyclodextrin treatment of murine Niemann-Pick C disease ameliorates neuronal cholesterol and glycosphingolipid storage and disease progression. PLoS ONE.

[35] Liu B. (2012). Therapeutic potential of cyclodextrins in the treatment of Niemann–Pick type C disease. Clin. Lipidol..

[36] Liu B., Turley S.D., Burns D.K., Miller A.M., Repa J.J., Dietschy J.M. (2009). Reversal of defective lysosomal transport in NPC disease ameliorates liver dysfunction and neurodegeneration in the npc1^−/−^ mouse. Proc. Natl. Acad. Sci. USA.

[37] Rosenbaum A.I., Zhang G., Warren J.D., Maxfield F.R. (2010). Endocytosis of beta-cyclodextrins is responsible for cholesterol reduction in Niemann-Pick type C mutant cells. Proc. Natl. Acad. Sci. USA.

[38] Taylor A.M., Liu B., Mari Y., Liu B., Repa J.J. (2012). Cyclodextrin mediates rapid changes in lipid balance in NPC1^−/−^ mice without carrying cholesterol through the bloodstream. J. Lipid Res..

[39] Vtesse. Inc. Study of 2-Hydroxypropyl-Beta-Cyclodextrin (vts-270) to Treat Niemann-Pick Type C1 (NPC1) Disease. Clinicaltrials. https://clinicaltrials.Gov/ct2/show/study/nct02534844.

[40] Aqul A., Liu B., Ramirez C.M., Pieper A.A., Estill S.J., Burns D.K., Liu B., Repa J.J., Turley S.D., Dietschy J.M. (2011). Unesterified cholesterol accumulation in late endosomes/lysosomes causes neurodegeneration and is prevented by driving cholesterol export from this compartment. J. Neurosci..

[41] Baldisseri D.M., Margolis J.W., Weber D.J., Koo J.H., Margolis F.L. (2002). Olfactory marker protein (OMP) exhibits a beta-clam fold in solution: Implications for target peptide interaction and olfactory signal transduction. J. Mol. Biol..

[42] Margolis F.L. (1982). Olfactory marker protein (OMP). Scand. J. Immunol..

[43] Arnold S.E., Lee E.B., Moberg P.J., Stutzbach L., Kazi H., Han L.Y., Lee V.M., Trojanowski J.Q. (2010). Olfactory epithelium amyloid-beta and paired helical filament-tau pathology in Alzheimer disease. Ann. Neurol..

[44] Attems J., Jellinger K.A. (2006). Olfactory tau pathology in Alzheimer disease and mild cognitive impairment. Clin. Neuropathol..

[45] Devanand D.P., Lee S., Manly J., Andrews H., Schupf N., Doty R.L., Stern Y., Zahodne L.B., Louis E.D., Mayeux R. (2015). Olfactory deficits predict cognitive decline and Alzheimer dementia in an urban community. Neurology.

[46] Berendse H.W., Booij J., Francot C.M., Bergmans P.L., Hijman R., Stoof J.C., Wolters E.C. (2001). Subclinical dopaminergic dysfunction in asymptomatic Parkinson’s disease patients’ relatives with a decreased sense of smell. Ann. Neurol..

[47] Hummel T., Witt M., Reichmann H., Welge-Luessen A., Haehner A. (2010). Immunohistochemical, volumetric, and functional neuroimaging studies in patients with idiopathic Parkinson’s disease. J. Neurol. Sci..

[48] Huard J.M., Youngentob S.L., Goldstein B.J., Luskin M.B., Schwob J.E. (1998). Adult olfactory epithelium contains multipotent progenitors that give rise to neurons and non-neural cells. J. Comp. Neurol..

[49] Kawagishi K., Ando M., Yokouchi K., Sumitomo N., Karasawa M., Fukushima N., Moriizumi T. (2015). Stereological estimation of olfactory receptor neurons in rats. Chem. Sens..

[50] Cluzeau C.V., Watkins-Chow D.E., Fu R., Borate B., Yanjanin N., Dail M.K., Davidson C.D., Walkley S.U., Ory D.S., Wassif C.A. (2012). Microarray expression analysis and identification of serum biomarkers for Niemann-Pick disease, type C1. Hum. Mol. Genet..

[51] Cataldo A.M., Paskevich P.A., Kominami E., Nixon R.A. (1991). Lysosomal hydrolases of different classes are abnormally distributed in brains of patients with Alzheimer disease. Proc. Natl. Acad. Sci. USA.

[52] Callahan L.M., Vaules W.A., Coleman P.D. (1999). Quantitative decrease in synaptophysin message expression and increase in cathepsin D message expression in Alzheimer disease neurons containing neurofibrillary tangles. J. Neuropathol. Exp. Neurol..

[53] Ginsberg S.D., Hemby S.E., Lee V.M., Eberwine J.H., Trojanowski J.Q. (2000). Expression profile of transcripts in Alzheimer’s disease tangle-bearing CA1 neurons. Ann. Neurol..

[54] Vitner E.B., Dekel H., Zigdon H., Shachar T., Farfel-Becker T., Eilam R., Karlsson S., Futerman A.H. (2010). Altered expression and distribution of cathepsins in neuronopathic forms of Gaucher disease and in other sphingolipidoses. Hum. Mol. Genet..

[55] Amritraj A., Wang Y., Revett T.J., Vergote D., Westaway D., Kar S. (2012). Role of Cathepsin D in U18666A-induced neuronal cell death: Potential implication in Niemann-Pick type C disease pathogenesis. J. Biol. Chem..

[56] Hovakimyan M., Maass F., Petersen J., Holzmann C., Witt M., Lukas J., Frech M.J., Hübner R., Rolfs A., Wree A. (2013). Combined therapy with cyclodextrin/allopregnanolone and miglustat improves motor but not cognitive functions in Niemann-Pick type C1 mice. Neuroscience.

[57] Hovakimyan M., Petersen J., Maass F., Reichard M., Witt M., Lukas J., Stachs O., Guthoff R., Rolfs A., Wree A. (2011). Corneal alterations during combined therapy with cyclodextrin/allopregnanolone and miglustat in a knock-out mouse model of npc1 disease. PLoS ONE.

[58] Tanaka Y., Yamada Y., Ishitsuka Y., Matsuo M., Shiraishi K., Wada K., Uchio Y., Kondo Y., Takeo T., Nakagata N. (2015). Efficacy of 2-hydroxypropyl-beta-cyclodextrin in Niemann-Pick disease type C model mice and its pharmacokinetic analysis in a patient with the disease. Biol. Pharm. Bull..

[59] Maarup T.J., Chen A.H., Porter F.D., Farhat N.Y., Ory D.S., Sidhu R., Jiang X., Dickson P.I. (2015). Intrathecal 2-hydroxypropyl-beta-cyclodextrin in a single patient with Niemann-Pick C1. Mol. Genet. Metab..

[60] Matsuo M., Togawa M., Hirabaru K., Mochinaga S., Narita A., Adachi M., Egashira M., Irie T., Ohno K. (2013). Effects of cyclodextrin in two patients with Niemann-Pick type C disease. Mol. Genet. Metab..

[61] Crumling M.A., Liu L., Thomas P.V., Benson J., Kanicki A., Kabara L., Halsey K., Dolan D., Duncan R.K. (2012). Hearing loss and hair cell death in mice given the cholesterol-chelating agent hydroxypropyl-beta-cyclodextrin. PLoS ONE.

[62] Ward S., O’Donnell P., Fernandez S., Vite C.H. (2010). 2-hydroxypropyl-beta-cyclodextrin raises hearing threshold in normal cats and in cats with Niemann-Pick type C disease. Pediatr. Res..

[63] Vite C.H., Bagel J.H., Swain G.P., Prociuk M., Sikora T.U., Stein V.M., O’Donnell P., Ruane T., Ward S., Crooks A. (2015). Intracisternal cyclodextrin prevents cerebellar dysfunction and purkinje cell death in feline Niemann-Pick type C1 disease. Sci. Transl. Med..

[64] Megias-Vericat J.E., Garcia-Robles A., Company-Albir M.J., Fernandez-Megia M.J., Perez-Miralles F.C., Lopez-Briz E., Casanova B., Poveda J.L. (2017). Early experience with compassionate use of 2 hydroxypropyl-beta-cyclodextrin for Niemann-Pick type C disease: Review of initial published cases. Neurol. Sci..

[65] Farbman A.I. (1990). Olfactory neurogenesis: Genetic or environmental controls?. Trends Neurosci..

[66] Hinds J.W., Hinds P.L., McNelly N.A. (1984). An autoradiographic study of the mouse olfactory epithelium: Evidence for long-lived receptors. Anat. Rec..

[67] Yuan J., Lipinski M., Degterev A. (2003). Diversity in the mechanisms of neuronal cell death. Neuron.

[68] Schlegel V., Thieme M., Holzmann C., Witt M., Grittner U., Rolfs A., Wree A. (2016). Pharmacologic treatment assigned for Niemann Pick type C1 disease partly changes behavioral traits in wild-type mice. Int. J. Mol. Sci..

[69] Davidson C.D., Fishman Y.I., Puskas I., Szeman J., Sohajda T., McCauliff L.A., Sikora J., Storch J., Vanier M.T., Szente L. (2016). Efficacy and ototoxicity of different cyclodextrins in Niemann-Pick C disease. Ann. Clin. Transl. Neurol..

[70] Hipler U.C., Schonfelder U., Hipler C., Elsner P. (2007). Influence of cyclodextrins on the proliferation of HaCaT keratinocytes in vitro. J. Biomed. Mater. Res. A.

[71] Yokoo M., Kubota Y., Motoyama K., Higashi T., Taniyoshi M., Tokumaru H., Nishiyama R., Tabe Y., Mochinaga S., Sato A. (2015). 2-hydroxypropyl-beta-cyclodextrin acts as a novel anticancer agent. PLoS ONE.

[72] Wang J.M., Johnston P.B., Ball B.G., Brinton R.D. (2005). The neurosteroid allopregnanolone promotes proliferation of rodent and human neural progenitor cells and regulates cell-cycle gene and protein expression. J. Neurosci..

[73] Zampieri S., Mellon S.H., Butters T.D., Nevyjel M., Covey D.F., Bembi B., Dardis A. (2009). Oxidative stress in Npc1 deficient cells: Protective effect of allopregnanolone. J. Cell. Mol. Med..

[74] Dover R., Patel K. (1994). Improved methodology for detecting bromodeoxyuridine in cultured cells and tissue sections by immunocytochemistry. Histochemistry.

[75] Onda K., Davis R.L., Shibuya M., Wilson C.B., Hoshino T. (1994). Correlation between the bromodeoxyuridine labeling index and the Mib-1 and Ki-67 proliferating cell indices in cerebral gliomas. Cancer.

